# Surgical outcomes of endoscopic thyroidectomy approaches for thyroid cancer: a systematic review and network meta-analysis

**DOI:** 10.3389/fendo.2023.1256209

**Published:** 2023-12-04

**Authors:** Xiaosong Li, Wei Ding, Hong Zhang

**Affiliations:** Department of Thyroid Surgery, The Second Hospital of Jilin University, Changchun, Jilin, China

**Keywords:** network meta-analysis, endoscopic thyroidectomy, conventional open thyroidectomy, thyroid cancer, surgical approaches

## Abstract

**Objectives:**

This network meta-analysis assesses the outcomes of seven endoscopic approaches, offering valuable insights for researchers and practitioners in choosing the best method for thyroid cancer patients.

**Methods:**

A systematic literature search was conducted in the PubMed, Embase and Web of Science databases up to March 2023. The analysis included seven endoscopic approaches, with a focus on their respective outcomes through network meta-analysis.

**Results:**

This meta-analysis included 44 studies involving 8,672 patients. The axillo-bilateral breast approach (ABBA) and unilateral axillo-breast approach (UABA) showed advantages in terms of reduced operative time compared to other approaches (MD = 19.66 minutes, 95% CI = -31.66 to 70.98; MD = 30.32 minutes, 95% CI = -1.45 to 62.09, respectively). The UABA and anterior chest approach (ACA) exhibited superiority in controlling intraoperative bleeding (MD = -3.37 mL, 95% CI = -22.58 to 15.85; MD = -13.77 mL, 95% CI = -28.85 1.31, respectively). UABA and ACA also showed advantages in reducing hospital stays (MD = -0.39 days, 95% CI = -1.48 to 0.71; MD = -0.26 days, 95% CI = -1.33 to 0.81, respectively). The transoral approach (OA) yielded results comparable to those of conventional open thyroidectomy (COT) and outperformed other endoscopic surgeries with regards to lymph node retrieval and metastatic lymph node assessment. For the stimulated serum thyroglobulin (TG) levels, no significant difference was observed between bilateral axillo-breast approach (BABA) and OA compared to COT. However, chest-breast approach (CBA) showed significantly lower levels than COT (MD=-0.40 ng/ml, 95% CI =-0.72 to -0.09). Patients in the gasless unilateral transaxillary approach (GUA) group experienced a significant improvement in cosmetic satisfaction (MD=-2.08, 95% CI =-3.35 to -0.82). Importantly, no significant difference was observed in the incidence of surgical complications between endoscopic thyroidectomy and COT.

**Conclusion:**

Endoscopic thyroid surgery is a safe and effective choice for thyroid cancer patients. Different approaches have their advantages, allowing personalized selection based on the patient’s needs. ABBA and UABA have shorter operative times, while UABA and ACA excel at controlling bleeding and shortening hospital stays. OA shows promise for lymph node assessment. These findings contribute to the growing evidence supporting endoscopic methods, expanding treatment options for thyroid cancer patients.

## 1 Introduction

Thyroid cancer, the most common endocrine-related cancer, has been increasing rapidly in recent years and is particularly prevalent in young women (1). Conventional open thyroidectomy (COT) is the standard surgical treatment for thyroid cancer; however, the conspicuous scarring and the associated emotional burden pose significant challenges, especially for young female patients. Endoscopic thyroidectomies were first performed in 1997 (2). This minimally invasive surgery not only provides better cosmetic results, but also contribute to reduces surgical complications. The introduction of endoscopic thyroidectomy has attracted the interest of a wide range of surgeons. Over the past 20 years, lumpectomies have developed rapidly. The most common surgical approaches are the gasless unilateral transaxillary approach (GUA), bilateral axillo-breast approach (BABA), axillo-bilateral breast approach (ABBA), unilateral axillo-breast approach (UABA), chest-breast approach (CBA), anterior chest approach (ACA) and transoral approach (OA).

It is important to acknowledge that endoscopic surgeries are not uniformly “minimally invasive” and do not universally reduce invasiveness. While they offer advantages in terms of cosmesis and surgical complication reduction, these advantages may not be consistently realized in every case. Endoscopic surgeries, while typically associated with reduced scarring and fewer complications, can, in certain circumstances, present unique challenges. For example, the use of unconventional entry sites can increase the risk of injury to critical nerves, such as brachial plexus injury with the axillary approach (3), or central nerve palsy with the transoral approach can lead to central nerve palsy (4). In light of these nuances and the potential variability in outcomes, a comprehensive evaluation of the efficacy and safety of endoscopic thyroid surgery is imperative. Previous meta-analyses have often compared different surgical approaches solely with COT, leaving critical gaps in our understanding. To address these limitations and provide a more nuanced assessment, we employ network meta-analysis (NMA). This approach integrates a wealth of clinical experiences, allowing us to offer a more balanced perspective on the advantages and disadvantages of endoscopic thyroid surgery across various surgical techniques. Network meta-analysis can showcase direct and indirect comparison results (5). Through NMA, we aim to gain a more comprehensive understanding of when and how endoscopic procedures can truly be minimally invasive and when they might not offer significant advantages over conventional approaches.

In summary, this network meta-analysis seeks to provide a comprehensive evaluation of endoscopic thyroid surgery, considering the variable nature of “minimally invasive” outcomes. By doing so, we aim to offer a more detailed perspective on the true merits and limitations of endoscopic surgery in the treatment of thyroid cancer.

## 2 Methods

Our systematic review was designed based on the Preferred Reporting Items for Systematic Reviews and Meta-Analyses(PROSPERO) guidelines (6) and registered with PROSPERO (CRD42023430413). Ethical approval or registration was not required as this study involved an analysis of existing published data.

### 2.1 Search strategy

The PubMed, Embase and Web of Science Core Collection (WoSCC) were searched up to March 28, 2023. The following keywords and comprehensive combinations thereof were included: (“endoscopic” OR “endoscopic surgery” OR “endoscopic-surgery”) AND (“thyroid cancer” OR “thyroid carcinoma”) AND (“thyroidectomy” OR “ thyroid surgery”).

### 2.2 Inclusion and exclusion criteria

The inclusion criteria were as follows: (1) Original English articles; (2) studies comparing endoscopic surgery with COT or different endoscopic surgical approaches; (3) studies reporting indicators such as surgical results or complications; and (4) studies in which the patients were diagnosed with thyroid cancer. The exclusion criteria were as follows: (1) letters, reviews, meeting abstracts, case reports, and editorials; (2) studies without control groups; and (3) studies that did not report original data.

### 2.3 Data extraction and quality assessment

Two researchers independently extracted the data. We documented study characteristics (the first author, publication year, surgery approach, country, study design, number of patients, age, extent of surgery and follow-up); surgical outcomes [operative time, intraoperative bleeding volume, hospital days, volume of drainage, duration of drainage, retrieved LNs, metastatic LNs, stimulated serum thyroglobulin (TG) levels and TG levels after postoperative radioactive iodine (RAI)], and surgical complications (transient hypocalcemia, permanent hypocalcemia, transient vocal cord palsy, permanent vocal cord palsy, transient hypoparathyroidism, permanent hypoparathyroidism, postoperative bleeding, chyle leakage, hematoma, seroma, recurrence and infection).

Two researchers independently evaluated the quality of the articles using the Newcastle-Ottawa Scale (NOS), and disagreements were settled through joint discussion. An article with an NOS score ≥ 6 was considered a high-quality study (7); the NOS scores of the articles we included were all > 6.

### 2.4 Statistical analysis

Data analysis was conducted using Stata14.1 software (Stata Corporation, College Station, TX, USA). Continuous variables (operative time, intraoperative bleeding volume, hospital days, volume of drainage, duration of drainage, retrieved LNs, metastatic LNs, stimulated TG levels, and TG levels after postoperative RAI) were recorded as mean differences (MD) with 95% confidence intervals (CI). Odds risks (OR) with 95% CI were calculated for dichotomous variables (transient hypocalcemia, permanent hypocalcemia, transient vocal cord palsy, permanent vocal cord palsy, transient hypoparathyroidism, permanent hypoparathyroidism, postoperative bleeding, chyle leakage, hematoma, seroma, recurrence, and infection). The consistency of the network was verified using local and global inconsistency tests. We used the node-splitting method to detect local inconsistencies between direct and indirect evaluations; If P< 0.05, local inconsistency was considered to exist. The surfaces under the cumulative ranking (SUCRA) curves and mean ranks were used to rank the eight surgical methods. The higher the SUCRA value, the higher the efficiency. We assessed potential publication bias by “comparison-adjusted” funnel plots.

## 3 Results

### 3.1 Study characteristics

**Figure 1:**
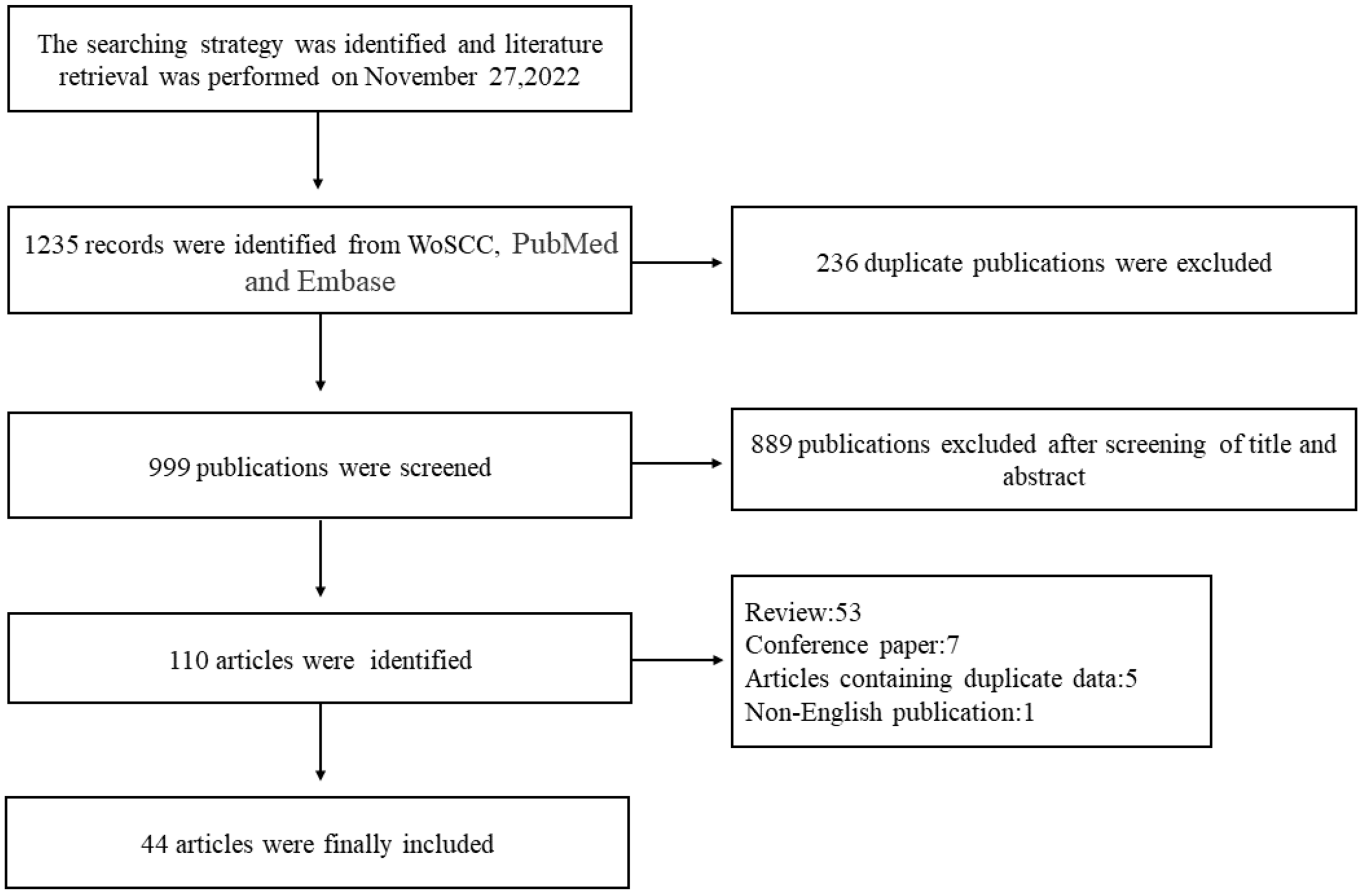
Flowchart of article screening. WoSCC, Web of Science Core Collection.

A total of 44 articles, involving a total of 8672 patients, were included in the NMA (**Figure 1**). Of these, 38 were retrospective studies and six were prospective studies. Details of the articles’ characteristics are listed in **Table 1**. Fifteen studies compared CBA with COT, five compared BABA with COT, eight compared OA with COT, two compared OA with CBA, nine compared GUA with COT, two compared ACA with COT, and two compared UABA with COT or CBA.

**Table 1:** Characteristics of meta-analysis included studies.

Author	Year	Country	Study design	No. of patients	Arm1	Arm2	N1	N2	F1	F2	Age1	Age2	Arm1 Extent of surgery	Arm2 Extent of surgery	Follow up
**Jong-hyuk Ahn (** 8)	2020	South Korea	R	275	OA	COT	150	125	145/150	89/125	43.06 ± 10.90	51.02 ± 12.42	Lob:110;TT:40	Lob:40;TT:85	median105(6-926)
**Bian Cong (** 9)	2018	China	R	60	OA	COT	30	30	29/30	30/30	26.50 ± 6.94	27.75 ± 6.94	N	N	N
**Min Ji Cho (** 10)	2015	South Korea	R	94	BABA	COT	49	45	47/49	36/45	39.39 ± 8.9	49.44 ± 12.3	Lob ± CND:16/21(76.2%);TT ± CND:33/73(45.2%)	Lob ± CND:5/21(23.8%);TT ± CND:40/74(54.8%)	1,447.73 ± 42.58
**Yoo Seung Chung (** 11)	2007	South Korea	R	301	BABA	COT	103	198	102/103	173/198	38.2 ± 8.2	47.2 ± 10.2	Lob:7;subTT:8;TT:88	Lob:12;subTT:14;TT:172	N
**Hengyuan Gao (** 12)	2019	China	R	102	CBA	COT	53	49	43/53	23/49	39.5 ± 11.6	46.5 ± 13.6	N	N	N
**Gong Yi (** 13)	2019	China	R	117	CBA	COT	72	45	50/72	21/45	36.4 ± 12.7	36.2 ± 11.6	N	N	20.6 ± 5.3
**Fangdong Guo (** 14)	2020	China	R	80	OA	CBA	40	40	40/40	40/40	29.8 ± 0.96	33.75 ± 1.19	N	N	N
**Youming Guo (** 15)	2018	China	R	38	CBA	COT	18	20	16/18	20/20	38.7 ± 11.8	43.0 ± 9.0	N	N	6 months
**Hyun Jun Hong (** 16)	2011	South Korea	R	117	GUA	COT	57	60	51/57	49/60	39.6 ± 7.88	41.77 ± 9.61	HT	HT	N
**Nitish Gupta (** 17)	2020	India	R	161	BABA	COT	40	121	37/40	108/60	38.69 ± 5.27	41.63 ± 6.20	TT;TT+CND	TT;TT+CND	>6 months
**Jian kang Huang (** 18)	2016	China	P	198	GUA	COT	75	123	59/75	92/123	37.8 ± 10.6	39.2 ± 11.3	N	N	52.4 ± 17.3
**Im Hyung-Jun (** 19)	2012	South Korea	R	46	BABA	COT	25	21	25/25	17/21	40.8 ± 7.2	49.7 ± 6.9	N	N	N
**Jong Ju Jeong (** 20)	2009	South Korea	R	449	GUA	COT	275	224	268/275	189/224	39.6 ± 8.8	49.5 ± 10.2	Lob:72;TT:203	Lob:9;TT:215	18.4(4-37)
**Eun Young Kim (** 21)	2017	South Korea	R	738	GUA	COT	200	538	192/200	400/538	39.5 ± 0.8	48.9 ± 0.5	TT	TT	N
**Seon Kwang Kim (** 22)	2015	South Korea	R	1003	ABBA	COT	173	830	160/173	734/173	37.95 ± 11.55	50.02 ± 19.34	Lob:116;subTT:1;TT:56	Lob:141;subTT:5;TT:684	69(52-77)
**Yoon Woo Koh (** 23)	2009	South Korea	R	59	GUA	COT	29	30	26/29	24/30	36.5 ± 5.1	38.3 ± 4.5	HT+CND	HT+CND	minimun 18months
**Wan Wook Kim (** 24)	2011	South Korea	R	228	BABA	COT	95	138	93/95	104/138	39.9 ± 9.1	51.8 ± 8.9	TT+CND	TT+CND	N
**Hayemin Lee (** 25)	2012	South Korea	R	78	UABA	COT	37	41	37/37	38/41	42.3 ± 7.6	49.0 ± 10.8	Lob	Lob	54.3
**Hongqiang Li (** 26)	2018	China	R	98	CBA	COT	46	52	35/46	38/52	37.0 ± 10.0	37.5 ± 11.0	HT:16;TT:30	HT:21;TT:31	N
**Tingting Li (** 27)	2022	China	P	172	GUA	COT	73	99	54/73	61/99	36.63 ± 7.48	38.91 ± 8.36	Lob+CND	Lob+CND	12months
**Peiliang Lin (** 28)	2020	China	R	91	ACA	COT	31	60	20/31	33/60	34.5 ± 10.98	44.75 ± 15.88	TT;subTT	TT;subTT	48 (19-76)
**Zhaodi Liu (** 29)	2020	China	R	135	OA	COT	59	76	49/59	47/76	30.4 ± 7.4	42.3 ± 8.6	Lob+CND:50;TT+CND:9	Lob+CND:30;TT+CND:46	32(15-36)
**Zhaodi Liu (** 29)	2020	China	R	102	CBA	COT	43	76	38/43	47/76	32.0 ± 7.9	42.3 ± 8.6	Lob+CND:30;TT+CND:13	Lob+CND:30;TT+CND:46	32(15-36)
**Zhaodi Liu (** 30)	2021	China	R	156	OA	COT	78	78	63/78	61/78	29.25 ± 2.04	31 ± 3.48	HT:57;TT:21	HT:54;TT:24	25 (19-34)
**KangNan Mo (** 31)	2018	China	R	55	UABA	CBA	22	33	21/22	32/33	31.7 ± 8.7	33.1 ± 7.8	HT:20;TT:2	HT:26;TT:7	N
**Ki Nam Park (** 32)	2015	South Korea	P	152	GUA	COT	50	102	46/50	88/102	38.0 ± 9.4	50.8 ± 11.5	TT	TT	1year
**Rui Qu (** 33)	2018	China	R	76	CBA	COT	40	36	31/40	22/36	36.7 ± 10.0	43.2 ± 14.3	TT ± CND	TT ± CND	N
**Yanqing Qu (** 34)	2021	China	R	134	CBA	COT	68	68	46/68	51/68	44.34 ± 9.82	45.56 ± 8.91	HT:37;TT:30	HT:34;TT:33	36.4 ± 6.3
**Xiaoting Ren (** 35)	2017	China	R	55	CBA	COT	20	35	20/20	35/35	36.05 ± 7.54	36.06 ± 5.65	Lob+CND	Lob+CND	N
**Luyi Si (** 36)	2022	China	R	62	CBA	UABA	26	36	18/26	33/36	34.00 ± 8.19	35.69 ± 7.66	HT+CND	HT+CND	N
**Haiqing Sun (** 4)	2022	China	P	92	OA	COT	28	56	27/28	54/56	36.57 ± 8.03	39.66 ± 8.67	TT+CND	TT+CND	16(3-54)
**Haiqing Sun (** 37)	2020	China	R	389	OA	COT	100	289	86/100	165/289	29.65 ± 6.57	45.18 ± 11.47	HT+CND	HT+CND	9(4-21)
**Haiqing Sun (** 37)	2020	China	R	408	CBA	COT	119	289	103/119	165/289	34.59 ± 7.69	45.18 ± 11.47	HT+CND	HT+CND	9(4-21)
**Peng Sun (** 38)	2022	China	R	590	CBA	COT	323	267	248/323	207/267	36.5 ± 10.7	45.6 ± 11.7	Lob+CND:172;TT+CND:151	Lob+CND:147;TT+CND:120	48.5 ± 14.4
**Kyung Tae (** 39)	2011	South Korea	R	67	GUA	COT	31	36	30/31	25/36	36.2 ± 9.9	44.6 ± 11.8	HT:28;TT:3	HT:36	36 ± 13
**Zhuo Tan (** 40)	2015	China	R	64	CBA	COT	34	30	32/34	26/30	30 ± 8.09	46.75 ± 14.73	HT+CND	HT+CND	N
**Denghuan Wang (** 41)	2022	China	P	40	OA	COT	20	20	14/20	14/20	33.2 ± 18.9	32.8 ± 19.1	HT+CND	HT+CND	N
**Tiantian Wang (** 42)	2020	China	P	160	OA	COT	80	80	80/80	80/80	31.48 ± 6.60	32.59 ± 5.18	N	N	22(12-33)
**Dapeng Xiang (** 43)	2020	China	R	96	CBA	COT	49	47	49/49	41/47	34.2 ± 7	46.9 ± 13.3	TT ± CNDTT+CND:43;TT+CND+CND:6	TT+CND:30;TT+CND:17	N
**Yang Yu (** 44)	2020	China	R	197	ACA	COT	85	112	72/85	74/112	38.15 ± 11.72	47.79 ± 10.51	HT:47;TT:38	HT:63;TT:49	N
**Yuquan Yuan (** 45)	2022	China	R	136	CBA	COT	63	73	N	N	32.51 ± 6.03	36.00 ± 8.01	HT+CND	HT+CND	N
**Daqi Zhang (** 46)	2019	China	R	400	CBA	COT	200	200	183/200	170/200	31.46 ± 7.75	37.28 ± 6.22	HT+CND;TT+CND	HT+CND;TT+CND	54 ± 3.4
**Weidong Zhang (** 47)	2021	China	R	95	OA	CBA	45	50	36/45	50/50	33.44 ± 6.87	34.44 ± 7.65	HT+CND	HT+CND	N
**Xing Zhang (** 48)	2023	China	R	545	CBA	GUA	263	282	233/263	262/282	42.2 ± 9.1	40.9 ± 7.7	HT+CND	HT+CND	21.2 ± 19.0
**Chuanming Zheng (** 49)	2018	China	R	22	GUA	COT	11	11	10/11	10/11	35.6 ± 2.6	48.5 ± 2.3	HT+CND	HT+CND	N
**Qunzai Zhao (** 50)	2018	China	R	306	CBA	COT	48	258	46/48	182/258	36.5 ± 8.5	47.1 ± 12.6	TT+CND	TT+CND	40.8 ± 4.7

TT, Total thyroidectomy; HT, hemithyroidectomy; subTT, subtotal thyroidectomy; Lob, lobectomy; CND, central neck lymph node dissection; GUA, gasless unilateral transaxillary approach; BABA=bilateral axillo-breast approach; ABBA, axillo-bilateral breast approach; UABA, unilateral axillo-breast approach; CBA, chest-breast approach; ACA, anterior chest approach; OA, transoral approach; COT, conventional open thyroidectomy.

### 3.2 Surgical outcomes

#### 3.2.1 Operative time (minute)

**Figure 2:**
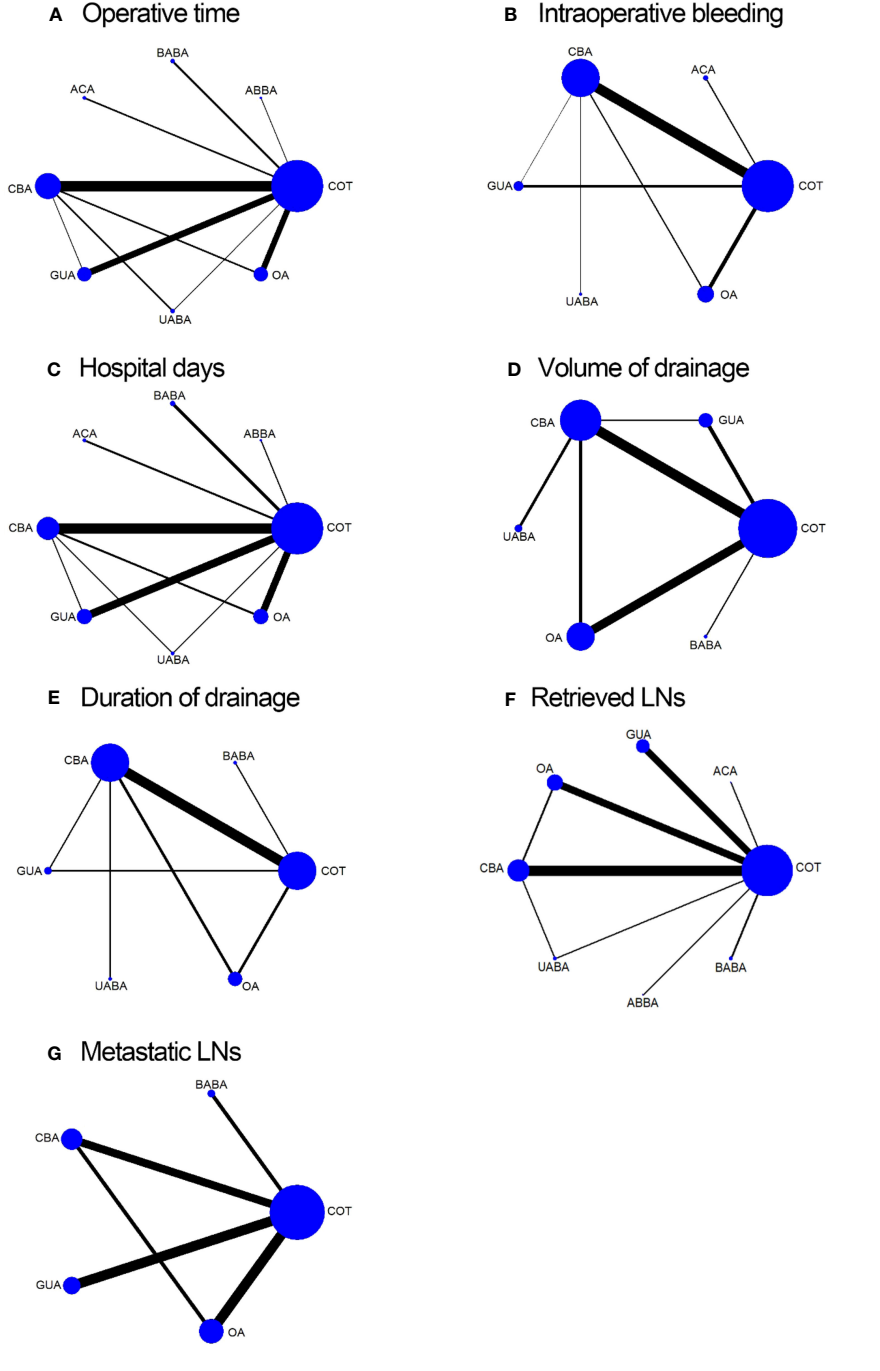
Network map of eligible comparisons for operative time **(A)**; intraoperative bleeding volume **(B)**; hospital days **(C)**; volume of drainage **(D)**; duration of drainage **(E)**; retrieved LNs **(F)**; metastatic LNs **(G)**.

Operative time was reported in 41 studies. A total of 8301 patients and eight surgical methods were included. The network relationships for different surgical modalities are shown in **Figure 2A**. The global inconsistency test was not significant (p= 0.7353), and a comparison of the estimated operative time of different surgical methods using NMA is shown in the **Table 2**. There was no significant difference (p>0.05) in operative time between the direct and indirect evaluations of the node-splitting models of local consistency. The shortest operative times were for the COT (95.6%), ABBA (71.5%), and UABA (61.4%), according to the SUCRA score.

**Table 2:** Network estimates of surgery comparisons for operative time and intraoperative bleeding volume.

COT	-	-3.37 (-22.58,15.85)	-13.77 (-28.85,1.31)	-	-0.07 (-5.13,4.99)	0.74 (-8.56,10.04)	3.14 (-4.08,10.35)
-19.66 (-70.98,31.66)	ABBA	–	–	–	–	–	–
-30.32 (-62.09,1.45)	-10.66 (-71.02,49.70)	UABA	-10.40 (-34.92,14.11)	–	3.30 (-15.24,21.84)	4.11 (-16.91,25.13)	6.50 (-13.63,26.64)
-33.25 (-69.87,3.38)	-13.59 (-76.63,49.46)	-2.92 (-51.41,45.56)	ACA	–	13.70 (-2.34,29.74)	14.51 (-3.29,32.31)	16.91 (0.05,33.77)
-38.02 (-68.29,-7.75)	-18.36 (-77.94,41.23)	-7.70 (-51.58,36.18)	-4.77 (-52.29,42.74)	BABA	–	–	–
-49.61 (-62.30,-36.92)	-29.95 (-82.82,22.92)	-19.29 (-50.24,11.66)	-16.36 (-55.13,22.40)	-11.59 (-44.42,21.24)	CBA	0.81 (-9.11,10.72)	3.20 (-4.66,11.07)
-50.60 (-67.21,-33.99)	-30.94 (-84.88,23.00)	-20.28 (-55.81,15.26)	-17.35 (-57.57,22.86)	-12.58 (-47.11,21.95)	-0.99 (-21.07,19.09)	GUA	2.40 (-9.18,13.97)
-77.18 (-93.81,-60.54)	-57.52 (-111.46,-3.57)	-46.85 (-82.10,-11.61)	-43.93 (-84.15,-3.71)	-39.16 (-73.70,-4.62)	-27.57 (-46.84,-8.29)	-26.58 (-49.93,-3.23)	OA

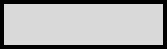
 operative time 
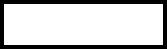
 intraoperative bleeding volume.

#### 3.2.2 Intraoperative bleeding (mL)

Twenty-five studies, involving a total of 4440 patients and six surgical methods, reported intraoperative bleeding volume. The network relationships between the different surgical methods are shown in **Figure 2B**. The global inconsistency test was not significant (p=0.6823), and the comparison of the NMA estimation of intraoperative bleeding volume between the different surgical methods is shown in **Table 2**. There was no significant difference (p>0.05) between the direct and indirect evaluations of the node-splitting models of local consistency for intraoperative bleeding volume. The surgical methods with the least amount of intraoperative bleeding were ACA (92.4%), UABA (57.1%), and COT (45.5%) according to the SUCRA score.

#### 3.2.3 Hospital days (days)

Hospital days were reported in 34 studies, involving a total of 7682 patients and a comparison of eight surgical methods. The network relationships between the different surgical methods are shown in **Figure 2C**. The global inconsistency test was not significant (p=0.6894), and the comparison of hospital days estimated by the NMA for the different surgical methods is shown in **Table S1**(Supplementary). No significant difference (p>0.05) was observed between the direct and indirect evaluations of the node-splitting models of local consistency for hospital days. The surgical procedures with the shortest hospital days were UABA (72.8%), ACA (66%), and COT (52.5%), according to the SUCRA score.

#### 3.2.4 Drainage situation

Twenty studies, involving a total of 3281 patients and the comparison of six surgical methods, recorded the volume of drainage. The network relationships between the different surgical methods are shown in **Figure 2D**. The global inconsistency test was not significant (p=0.2120). A comparison of the NMA estimation for different surgical methods is shown in **Table S2** (Supplementary). There was no significant difference (p>0.05) between the direct and indirect evaluations of the node-splitting models of local consistency for volume of drainage. The surgical methods with the lowest volume of drainage were COT (92.1%), UABA (65.7%), and BABA (61.1%), according to the SUCRA score. Fourteen studies, involving a total of 2238 patients and the comparison of six surgical methods, recorded the duration of drainage. The network relationships between the different surgical methods are shown in **Figure 2E**. The global inconsistency test was not significant (p=0.8308). A comparison of the duration of drainage estimated by NMA for different surgical methods is shown in **Table S3**. There was no significant difference (p>0.05) between the direct and indirect evaluations of the node-splitting models of local consistency for duration of drainage. The shortest surgical procedures for the duration of drainage were COT (76.9%), UABA (68.2%), and OA (50.2%), according to the SUCRA score.

#### 3.2.5 Lymph node situation

The number of retrieved LNs was reported in 33 studies, involving a total of 6569 patients and eight surgical methods. The network relationship between the number of retrieved LNs and the different surgical methods is shown in **Figure 2F**. The global inconsistency test was not significant (p=0.2835), and a comparison of the number of lymph node recoveries estimated by the NMA for different surgical methods is shown in **Table S4**(Supplementary). There was no significant difference (p>0.05) between the direct and indirect evaluations of the node-splitting models of local consistency for the number of retrieved LNs. The surgical methods with the highest number of retrieved LNs were ACA (81.4%), COT (72.7%), and OA (71.6%) according to the SUCRA grade. Seventeen studies discussed the number of metastatic LNs, involving a total of 3453 patients and comparing five surgical methods. The network relationship between the number of metastatic LNs and different surgical methods is shown in **Figure 2G**. The global inconsistency test was not significant (p=0.3129), and a comparison of the number of metastatic LNs estimated by NMA for different surgical methods is shown in **Table S5** (Supplementary). There was no significant difference (p>0.05) between the direct and indirect evaluations of the node-splitting models of local consistency for the number of metastatic LNs. The surgical methods with the highest number of metastatic LNs were OA (63.3%), BABA (61.4%), and COT (54.9%), according to the SUCRA grade.

### 3.3 Stimulated TG levels and TG levels after RAI

In our meta-analysis, we found that stimulated TG levels were not significantly different between BABA (MD=0.04 ng/ml,[-0.41,0.49], *p* =0.86), OA (MD=-0.46 ng/ml,[-1.39,0.48],*p* =0.34), and COT groups. However, it was significantly lower in the CBA group (MD=-0.40 ng/ml,[-0.72,-0.09];*p* =0.01) than in the COT group (**Figure S1** in Supplementary). There was no significant difference in TG levels after RAI treatment between the CBA (MD=-0.13[-0.31,0.06],*p* =0.18)and COT groups (**Figure S2** in Supplementary).

### 3.4 Postoperative pain

Five studies reported postoperative pain using the visual analog scale (VAS); detailed information is shown in the **Figure S3** (Supplementary). Our meta-analysis showed that CBA was not significantly lower than COT in terms of postoperative pain (MD=-0.66[-1.39,0.07], *p* =0.08).

### 3.5 Cosmetic satisfaction

There have been reports of postoperative cosmetic satisfaction with the common measurement method, detailed information was shown in **Figure S4** (Supplementary). Our meta-analysis showed that GUA was significantly better than COT in terms of cosmetic satisfaction (MD=-2.08[-3.35,-0.82], *p* =0.001).

### 3.6 Surgical complications

We compared the incidence of bleeding, transient or permanent hypoparathyroidism, transient or permanent vocal cord palsy, transient or permanent hypocalcemia, chyle leak, infection, seroma, hematoma, and recurrence between the endoscopic and COT groups. Our results suggested no significant difference in the incidence of surgical complications between the two groups (**Table 3**).

**Table 3:** Comparison of endoscopic thyroidectomy and COT for surgical complications.

Complication	Surgery	Model Selected	OR (95% CI)	*P*
**Bleeding**	BABA	Fixed	1.76(0.45,6.85)	0.42
	GUA	Fixed	1.91(0.51,7.20)	0.34
**transient hypoparathyroidism**	OA	Random	0.55(0.25,1.25)	0.16
	CBA	Random	2.34(0.74,7.37)	0.15
**permanent hypoparathyroidism**	OA	Fixed	0.35 (0.07,1.91)	0.23
	CBA	Fixed	1.45(0.55,3.78)	0.45
**transient vocal cord palsy**	OA	Fixed	0.55(0.29,1.04)	0.07
	CBA	Fixed	0.90(0.53,1.53)	0.69
	GUA	Fixed	1.50(0.97,2.32)	0.07
	BABA	Random	2.99(0.97,9.19)	0.06
**permanent vocal cord palsy**	OA	Fixed	1.76(0.16,18.95)	0.64
	GUA	Fixed	1.30(0.39,4.32)	0.67
	BABA	Fixed	5.33(0.53,56.79)	0.16
**transient hypocalcemia**	BABA	Fixed	1.13(0.79,1.62)	0.51
	CBA	Fixed	0.52(0.27,1.01)	0.05
	GUA	Random	1.20(0.42,3.47)	0.74
**permanent hypocalcemia**	BABA	Fixed	0.41(0.14,1.26)	0.12
	GUA	Fixed	0.51(0.15,1.76)	0.29
**chyle leak**	CBA	Fixed	0.26(0.06,1.14)	0.07
	OA	Random	1.12(0.07,19.39)	0.94
**Infection**	CBA	Fixed	0.43(0.08,2.38)	0.34
	OA	Fixed	1.55(0.22,10.88)	0.66
**Seroma**	GUA	Fixed	1.98(0.99,3.96)	0.05
	BABA	Fixed	1.08(0.21,5.56)	0.93
**Hematoma**	CBA	Fixed	1.13(0.24,5.29)	0.88
	GUA	Fixed	0.65(0.11,3.93)	0.64
**Recurrence**	CBA	Fixed	1.74(0.40,7.48)	0.46
	GUA	Fixed	0.25(0.03,2.08)	0.2

### 3.7 Ranking

**Figure 3:**
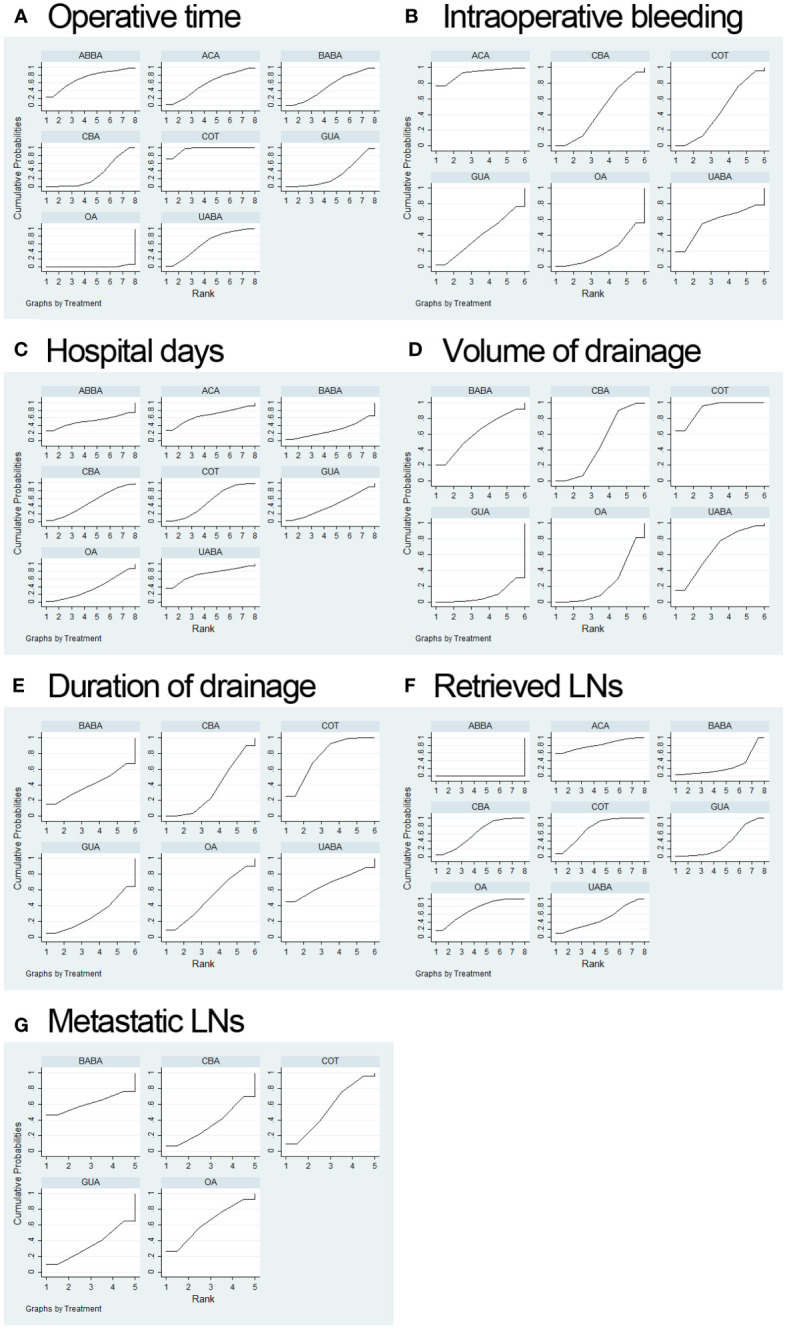
Surgery ranking curves for operative time **(A)**; intraoperative bleeding volume **(B)**; hospital days **(C)**; volume of drainage **(D)**; duration of drainage **(E)**; retrieved LNs **(F)**; metastatic LNs **(G)**.

Our NMA ranked various surgical methods for operative time, intraoperative bleeding volume, hospital days, volume of drainage, duration of drainage, retrieved LNs and metastatic LNs (**Figure 3**).

### 3.8 Publication bias

The comparison-adjusted funnel plots of this meta-analysis clearly showed that the included studies were symmetrical, indicating a relatively low publication bias in terms of operative time, intraoperative bleeding volume, hospital days, volume of drainage, duration of drainage, retrieved LNs, metastatic LNs. Detailed information is provided in the **Figure S5** (Supplementary).

## 4 Discussion

This meta-analysis found that endoscopic surgery not only has a lower incidence of complications than COT but may even be superior to COT in terms of operative bleeding volume, hospital days, and retrieved LNs. Previous meta-analyses have explored the efficacy of endoscopic thyroidectomy for thyroid neoplasms, but only compared different endoscopic surgeries or conventional surgeries (51, 52). Our study adopted an NMA approach to comprehensively and meticulously discuss the advantages of seven surgical approaches. Among the various surgical approaches, ABBA stands out for its notably shorter operative time and proximity to COT. ACA excels in effectively controlling intraoperative bleeding. UABA demonstrates superior outcomes in terms of hospital stay and drainage, while OA prove to be more conducive to straightforward lymph node acquisition. The patients included in the study were all patients with thyroid cancer, and studies only focused on different endoscopic surgeries, which greatly reduced research heterogeneity and made our results more reliable.

COT required the least operative time, which is consistent with previous research results (15, 16, 22). This may be because of the need for additional surgical channels and workspaces for endoscopic surgery. For endoscopic surgery, the ABBA and UABA require shorter operative times. It needs to be emphasized that OA serves as the “shortest surgical pathway” for endoscopic surgery, but the results show that it has the longest operative time. The main reason for this phenomenon may be: 1) To improve safety and feasibility, OA usually involves three oral vestibular incisions (53), and during the surgical process, the three trocars often encounter unavoidable collisions in the limited workspace, subsequently increasing the difficulty of the surgery; 2) OA is a top-down operation. Owing to the presence of the thyroid cartilage, treatment of the superior thyroid requires more time than other methods. Many of the operation time data included in this study did not specify whether it was a total thyroidectomy or lobectomy, and roughly categorizing them into the same category would, to some extent, have affected our conclusion. ACA and UABA show obvious advantages in terms of intraoperative bleeding volume and hospital stay, while OA and GUA often have poor control of intraoperative bleeding volume due to the need for more extensive free platysma muscle to create surgical space. In the transaxillary approach, the UABA is relatively better than the GUA in bleeding control; however, because it does not require CO_2_ insufflation, it avoids the occurrence of mediastinal emphysema, subcutaneous emphysema, and other related gas complications.

Thyroid cancer has a high rate of central lymph node metastasis (54), which is associated with high patient mortality (55). This study reveals that ACA outperforms COT in terms of retrieving LNs; however, it’s important to note that the limited inclusion of only two articles involving ACA may introduce potential bias into the interpretation of this result. OA exhibits superior results in the retrieval of both total LNs and metastatic LNs compared to other endoscopic procedures. This can be attributed to the fact that, from top to bottom, the field of view in OA can seamlessly encompass the central region, thereby circumventing sternum and clavicle limitations, which uniquely facilitates the removal of central LNs.

TG is an important indicator for the postoperative follow-up of thyroid cancer, and lower stimulated TG levels are considered to be associated with total thyroidectomy and low recurrence rate (56). We found no difference between the endoscopic surgery and COT groups for TG, with CBA being significantly lower than COT. Regarding postoperative pain, CBA was not significantly better than COT. The reasons for this may be as follows: (1) fewer articles were included and (2) inconsistent measurement time points after surgery. We found that many studies used different methods to measure postoperative pain, which made it difficult to merge the results of the studies. However, this meta-analysis included studies that used VAS scores. It is well known that endoscopic thyroid surgery avoids scarring on the neck, and our results fully demonstrate this advantage. However, we also encountered a difficulty, there is no common scoring criterion for objective evaluation, and it is necessary for us to find a more objective standard in future research. The incidence of postoperative complications was not lower in the endoscopic surgery group than that in the COT group. Each surgical approach has its own drawbacks; for example, OA is prone to incision infection (8), CBA causes subcutaneous effusion (57), and GUA causes brachial plexus nerve injury (58).

Our NMA had some limitations. First, most of the included articles were retrospective studies, and further large-scale randomized controlled trials are needed. Second, the articles we included were all from Asia, which may limit the generalisability of our results. Third, the scope of surgery included in the article was not specified, and there was a certain degree of heterogeneity.

## 5 Conclusion

Based on comprehensive NMA, endoscopic thyroidectomy is safe and feasible. Endoscopic surgical approaches have unique advantages. ABBA and UABA are the closest to COT in terms of operative time and are superior to other endoscopic surgeries. The UABA and ACA are superior to other surgical approaches in controlling intraoperative bleeding and hospital stay. UABA and BABA showed significant advantages in terms of postoperative drainage volume. The OA is more adept at retrieving lymph nodes. Endoscopic thyroidectomy did not increase the incidence of postoperative complications. However, it is clear that careful selection and consideration of the surgical approach are essential. In light of these findings, it is apparent that prospective, randomized studies are needed to validate and further refine the findings of our NMA, providing a more robust foundation for surgeons to make informed decisions when selecting appropriate endoscopic surgical approaches.

## Author contributions

XL: Data curation, Writing – original draft. HZ: Writing – review & editing. WD: Data curation, Writing – original draft.
