# Supplementary material for: Surgical outcomes of endoscopic thyroidectomy approaches for thyroid cancer: a systematic review and network meta-analysis
Source: Front Endocrinol (Lausanne). 2023 Dec 4;14:1256209. doi: 10.3389/fendo.2023.1256209 (PMC10726028; doi:10.3389/fendo.2023.1256209)
Supplement: Supplementary file 1 [file DataSheet_1.pdf]

Figure S1. Forest plots of stimulated TG levels for BABA, OA and CBA.

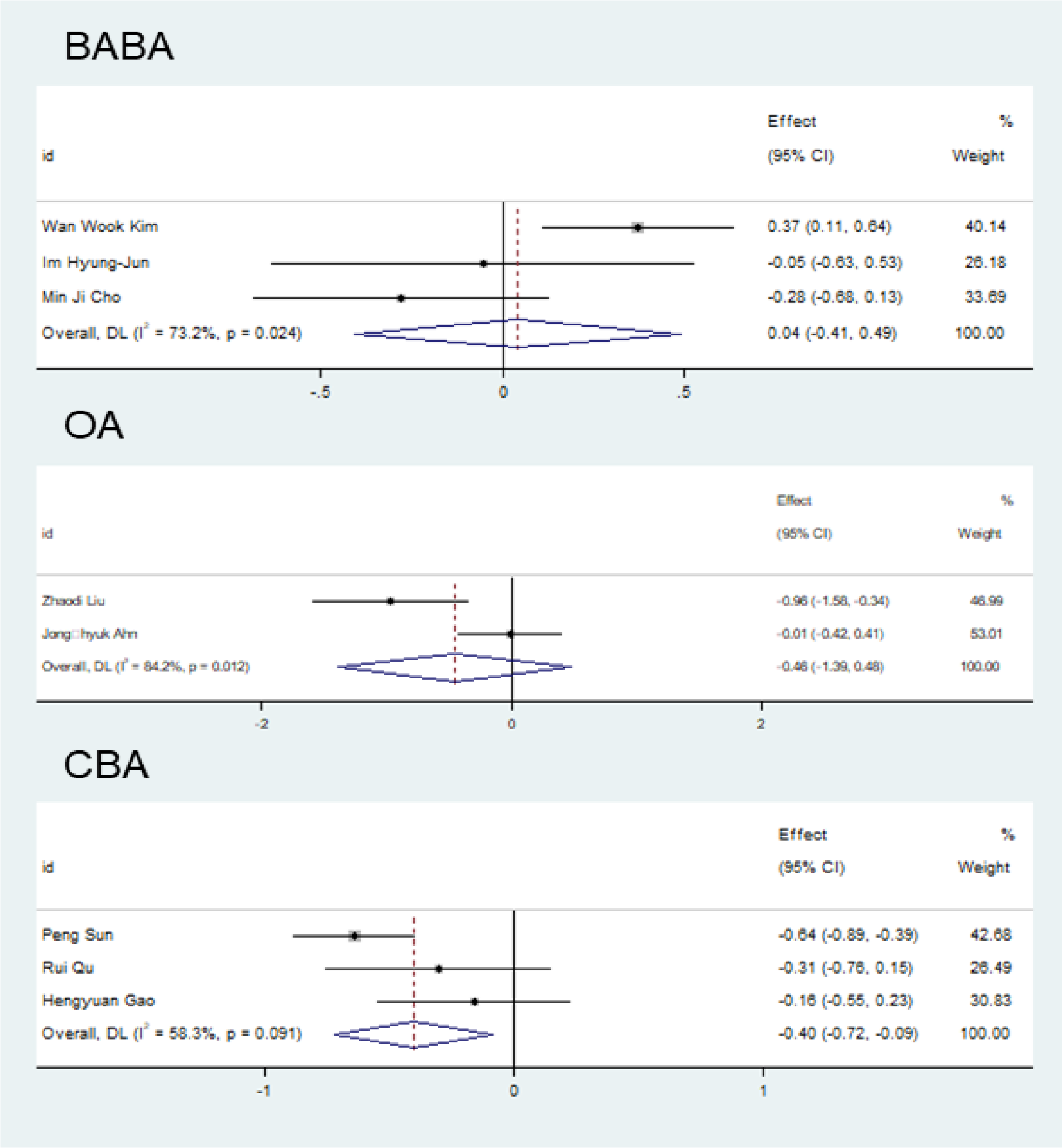

Figure S2. Forest plots of TG levels after RAI treatment for CBA.

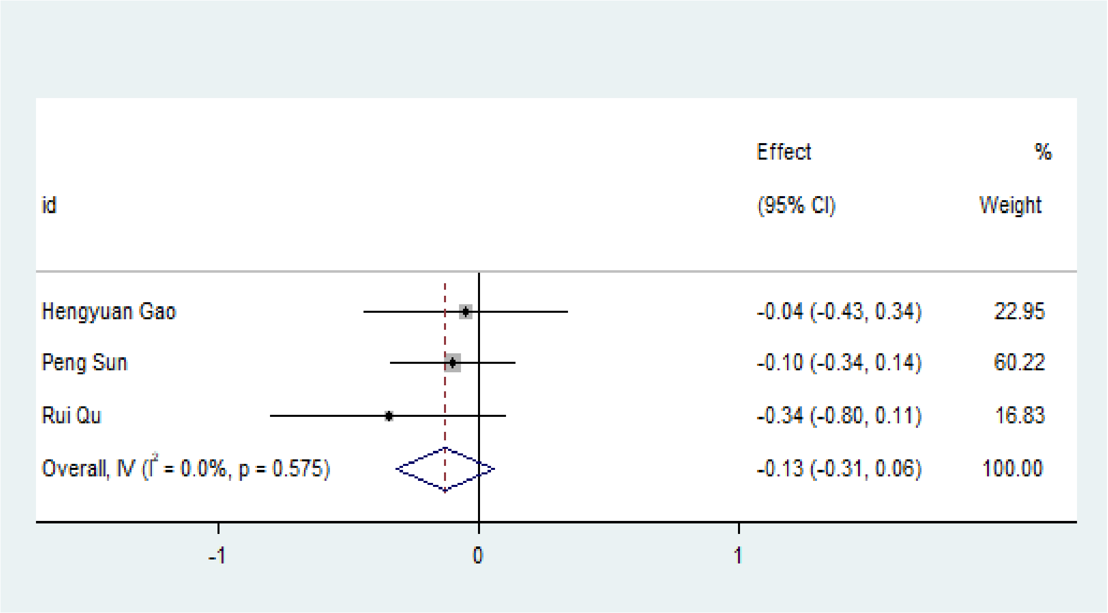

Figure S3. Forest plots of postoperative pain for CBA.

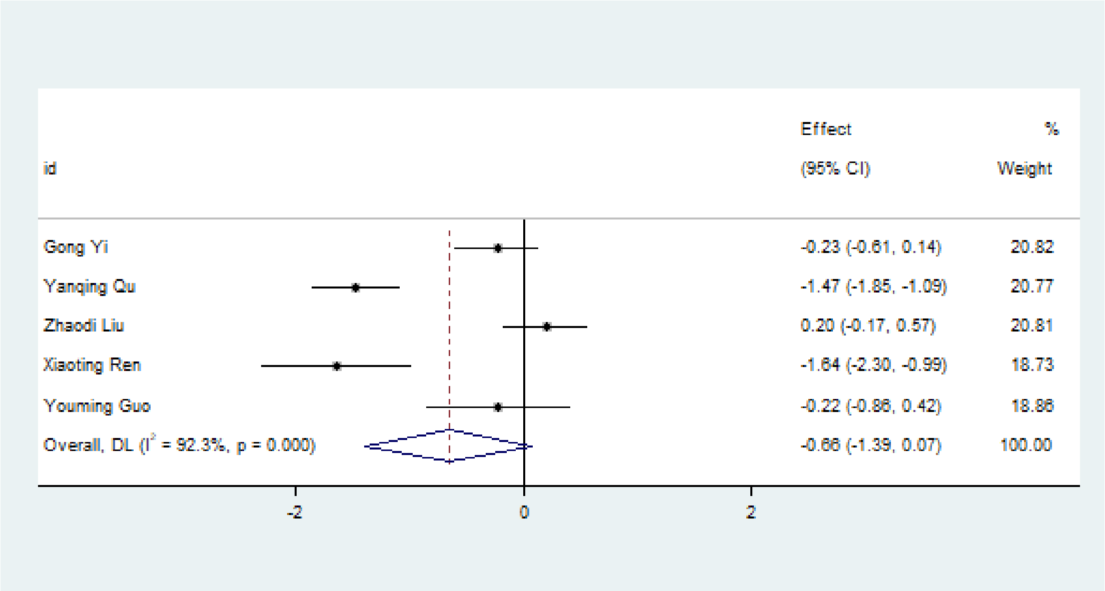

Figure S4. Forest plots of cosmetic satisfaction for GUA.

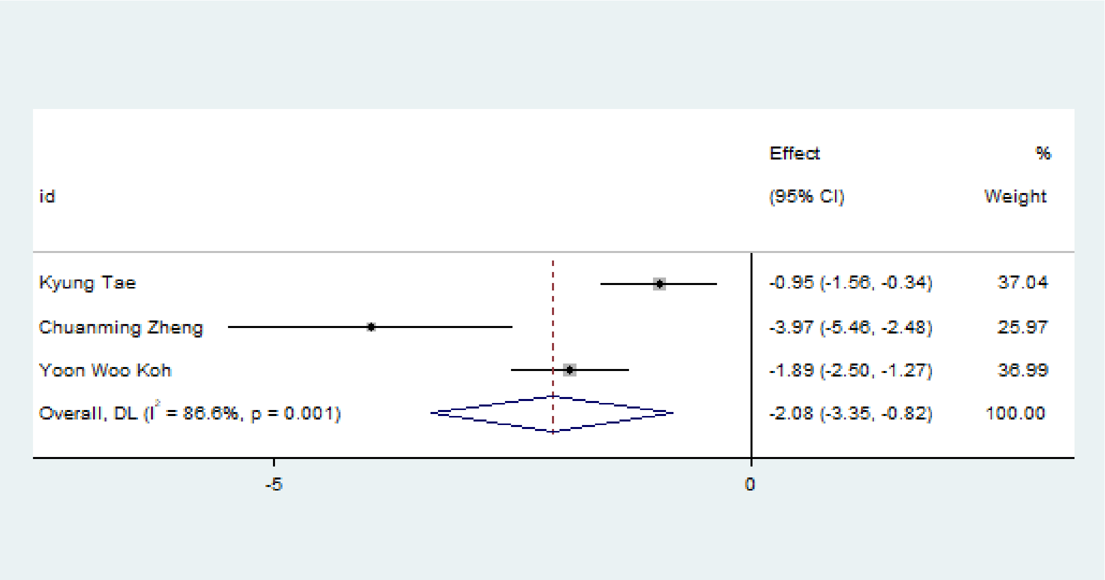

Figure S5. Funnel plots for operative time(a), intraoperative bleeding volume(b), hospital days(c), volume of drainage(d), duration of drainage(e), retrieved LNs(f) and metastatic LNs(g).

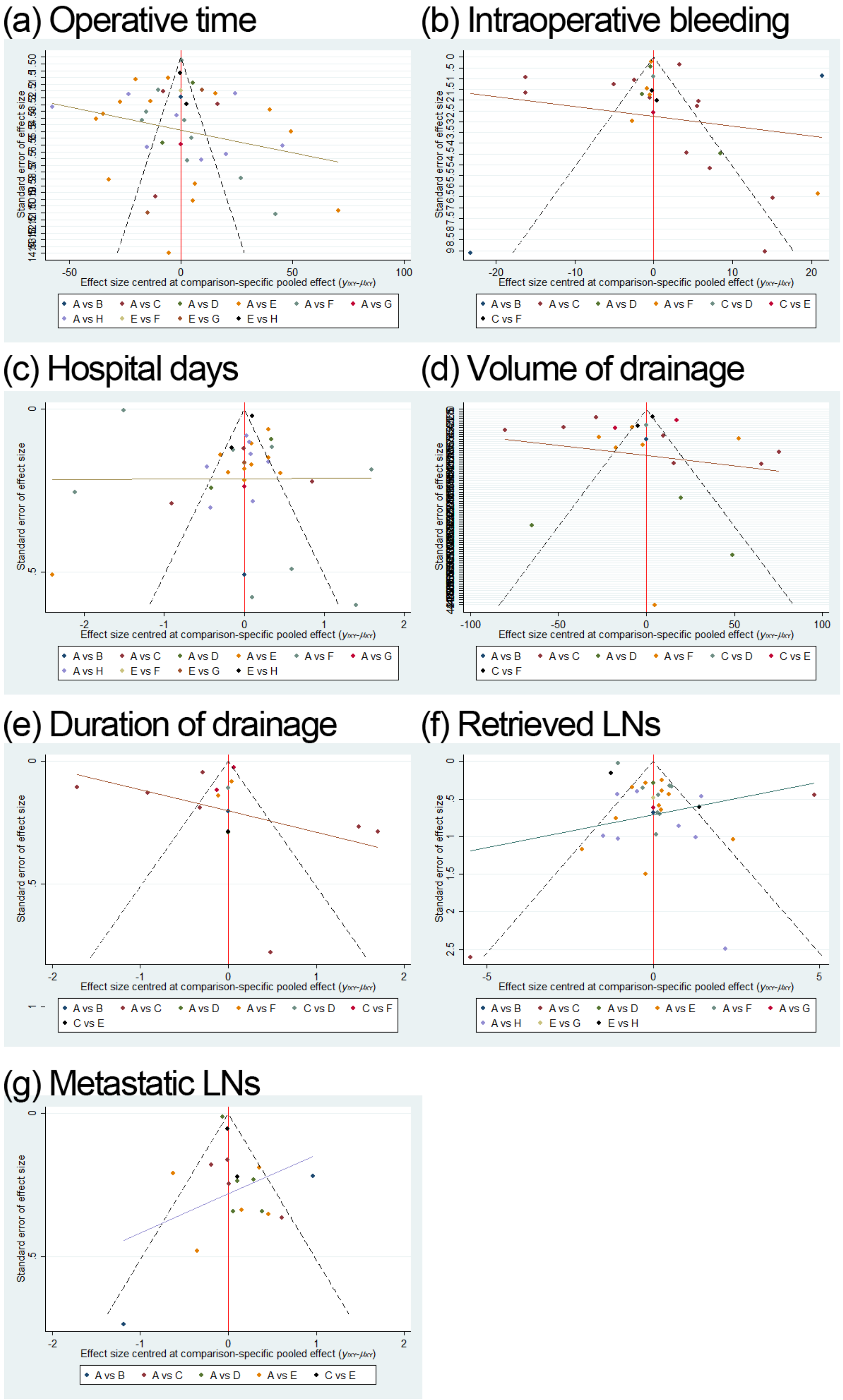

(a) Operative time
Standard error of effect size
Effect size centred at comparison-specific pooled effect ($y_{ixy}-\mu_{xy}$)
-50
0
50
100
A vs B
A vs C
A vs D
A vs E
A vs F
A vs G
A vs H
E vs F
E vs G
E vs H
(b) Intraoperative bleeding
Standard error of effect size
Effect size centred at comparison-specific pooled effect ($y_{ixy}-\mu_{xy}$)
-20
-10
0
10
20
A vs B
A vs C
A vs D
A vs F
C vs D
C vs E
C vs F
(c) Hospital days
Standard error of effect size
0
.5
Effect size centred at comparison-specific pooled effect ($y_{ixy}-\mu_{xy}$)
-2
-1
0
1
2
A vs B
A vs C
A vs D
A vs E
A vs F
A vs G
A vs H
E vs F
E vs G
E vs H
(d) Volume of drainage
Standard error of effect size
Effect size centred at comparison-specific pooled effect ($y_{ixy}-\mu_{xy}$)
-100
-50
0
50
100
A vs B
A vs C
A vs D
A vs F
C vs D
C vs E
C vs F
(e) Duration of drainage
Standard error of effect size
0
.5
1
Effect size centred at comparison-specific pooled effect ($y_{ixy}-\mu_{xy}$)
-2
-1
0
1
2
A vs B
A vs C
A vs D
A vs F
C vs D
C vs F
C vs E
(f) Retrieved LNs
Standard error of effect size
0
.5
1
1.5
2
2.5
Effect size centred at comparison-specific pooled effect ($y_{ixy}-\mu_{xy}$)
-5
0
5
A vs B
A vs C
A vs D
A vs E
A vs F
A vs G
A vs H
E vs G
E vs H
(g) Metastatic LNs
Standard error of effect size
0
.5
Effect size centred at comparison-specific pooled effect ($y_{ixy}-\mu_{xy}$)
-2
-1
0
1
2
A vs B
A vs C
A vs D
A vs E
C vs E

Table S1. Network estimates of surgery comparisons for hospital days.

UABA	0.13 (-1.40,1.66)	0.39 (-0.71,1.48)	0.35 (-1.74,2.43)	0.40 (-0.69,1.50)	0.48 (-0.73,1.69)	0.53 (-0.66,1.73)	0.69 (-0.72,2.09)
-0.13 (-1.66,1.40)	ACA	0.26 (-0.81,1.33)	0.22 (-1.85,2.29)	0.27 (-0.87,1.42)	0.35 (-0.84,1.55)	0.41 (-0.78,1.59)	0.56 (-0.83,1.94)
-0.39 (-1.48,0.71)	-0.26 (-1.33,0.81)	COT	-0.04 (-1.81,1.73)	0.02 (-0.41,0.44)	0.09 (-0.44,0.63)	0.15 (-0.36,0.66)	0.30 (-0.58,1.18)
-0.35 (-2.43,1.74)	-0.22 (-2.29,1.85)	0.04 (-1.73,1.81)	ABBA	0.06 (-1.77,1.88)	0.13 (-1.72,1.99)	0.19 (-1.66,2.03)	0.34 (-1.64,2.32)
-0.40 (-1.50,0.69)	-0.27 (-1.42,0.87)	-0.02 (-0.44,0.41)	-0.06 (-1.88,1.77)	CBA	0.08 (-0.57,0.73)	0.13 (-0.46,0.73)	0.28 (-0.70,1.26)
-0.48 (-1.69,0.73)	-0.35 (-1.55,0.84)	-0.09 (-0.63,0.44)	-0.13 (-1.99,1.72)	-0.08 (-0.73,0.57)	GUA	0.06 (-0.68,0.79)	0.21 (-0.83,1.24)
-0.53 (-1.73,0.66)	-0.41 (-1.59,0.78)	-0.15 (-0.66,0.36)	-0.19 (-2.03,1.66)	-0.13 (-0.73,0.46)	-0.06 (-0.79,0.68)	OA	0.15 (-0.87,1.17)
-0.69 (-2.09,0.72)	-0.56 (-1.94,0.83)	-0.30 (-1.18,0.58)	-0.34 (-2.32,1.64)	-0.28 (-1.26,0.70)	-0.21 (-1.24,0.83)	-0.15 (-1.17,0.87)	BABA

Table S2. Network estimates of surgery comparisons for volume of drainage.

COT	32.45 (-37.32,102.23)	35.40 (-54.51,125.31)	56.00 (26.24,85.76)	78.39 (44.74,112.03)	100.82 (50.73,150.91)
-32.45 (-102.23,37.32)	UABA	2.95 (-110.86,116.75)	23.55 (-39.56,86.65)	45.93 (-28.39,120.26)	68.37 (-14.31,151.04)
-35.40 (-125.31,54.51)	-2.95 (-116.75,110.86)	BABA	20.60 (-74.10,115.30)	42.99 (-53.01,138.98)	65.42 (-37.50,168.34)
-56.00 (-85.76,-26.24)	-23.55 (-86.65,39.56)	-20.60 (-115.30,74.10)	CBA	22.39 (-16.88,61.65)	44.82 (-8.59,98.23)
-78.39 (-112.03,-44.74)	-45.93 (-120.26,28.39)	-42.99 (-138.98,53.01)	-22.39 (-61.65,16.88)	OA	22.43 (-36.67,81.54)
-100.82 (-150.91,-50.73)	-68.37 (-151.04,14.31)	-65.42 (-168.34,37.50)	-44.82 (-98.23,8.59)	-22.43 (-81.54,36.67)	GUA

Table S3. Network estimates of surgery comparisons for duration of drainage.

COT	-0.09 (-2.18,1.99)	0.39 (-0.62,1.40)	0.64 (-1.29,2.57)	0.61 (-0.07,1.28)	0.84 (-0.57,2.26)
0.09 (-1.99,2.18)	UABA	0.48 (-1.73,2.70)	0.73 (-2.11,3.58)	0.70 (-1.27,2.67)	0.93 (-1.49,3.36)
-0.39 (-1.40,0.62)	-0.48 (-2.70,1.73)	OA	0.25 (-1.93,2.43)	0.22 (-0.79,1.22)	0.45 (-1.22,2.12)
-0.64 (-2.57,1.29)	-0.73 (-3.58,2.11)	-0.25 (-2.43,1.93)	BABA	-0.03 (-2.08,2.02)	0.20 (-2.19,2.60)
-0.61 (-1.28,0.07)	-0.70 (-2.67,1.27)	-0.22 (-1.22,0.79)	0.03 (-2.02,2.08)	CBA	0.23 (-1.18,1.64)
-0.84 (-2.26,0.57)	-0.93 (-3.36,1.49)	-0.45 (-2.12,1.22)	-0.20 (-2.60,2.19)	-0.23 (-1.64,1.18)	GUA

Table S4. Network estimates of surgery comparisons for retrieved LNs.

ACA	-0.44 (-2.10,1.22)	-0.42 (-2.21,1.37)	-0.58 (-2.33,1.16)	-0.87 (-3.03,1.28)	-1.05 (-2.83,0.74)	-1.68 (-4.13,0.77)	-12.37 (-15.01,-9.73)
0.44 (-1.22,2.10)	COT	0.02 (-0.66,0.70)	-0.14 (-0.68,0.40)	-0.43 (-1.81,0.94)	-0.61 (-1.25,0.04)	-1.24 (-3.05,0.56)	-11.93 (-13.99,-9.87)
0.42 (-1.37,2.21)	-0.02 (-0.70,0.66)	OA	-0.16 (-0.93,0.60)	-0.45 (-1.96,1.05)	-0.63 (-1.56,0.31)	-1.26 (-3.20,0.67)	-11.95 (-14.12,-9.78)
0.58 (-1.16,2.33)	0.14 (-0.40,0.68)	0.16 (-0.60,0.93)	CBA	-0.29 (-1.66,1.08)	-0.46 (-1.31,0.38)	-1.10 (-2.97,0.77)	-11.79 (-13.92,-9.66)
0.87 (-1.28,3.03)	0.43 (-0.94,1.81)	0.45 (-1.05,1.96)	0.29 (-1.08,1.66)	UABA	-0.17 (-1.69,1.35)	-0.81 (-3.08,1.46)	-11.50 (-13.97,-9.02)
1.05 (-0.74,2.83)	0.61 (-0.04,1.25)	0.63 (-0.31,1.56)	0.46 (-0.38,1.31)	0.17 (-1.35,1.69)	GUA	-0.64 (-2.56,1.29)	-11.32 (-13.48,-9.17)
1.68 (-0.77,4.13)	1.24 (-0.56,3.05)	1.26 (-0.67,3.20)	1.10 (-0.77,2.97)	0.81 (-1.46,3.08)	0.64 (-1.29,2.56)	BABA	-10.69 (-13.42,-7.95)
12.37 (9.73,15.01)	11.93 (9.87,13.99)	11.95 (9.78,14.12)	11.79 (9.66,13.92)	11.50 (9.02,13.97)	11.32 (9.17,13.48)	10.69 (7.95,13.42)	ABBA

Table S5. Network estimates of surgery comparisons for metastatic LNs.

OA	0.03 (-0.66,0.73)	-0.04 (-0.32,0.24)	-0.11 (-0.52,0.29)	-0.11 (-0.41,0.20)
-0.03 (-0.73,0.66)	BABA	-0.07 (-0.70,0.56)	-0.15 (-0.86,0.57)	-0.14 (-0.85,0.56)
0.04 (-0.24,0.32)	0.07 (-0.56,0.70)	COT	-0.08 (-0.38,0.22)	-0.07 (-0.35,0.21)
0.11 (-0.29,0.52)	0.15 (-0.57,0.86)	0.08 (-0.22,0.38)	GUA	0.01 (-0.40,0.41)
0.11 (-0.20,0.41)	0.14 (-0.56,0.85)	0.07 (-0.21,0.35)	-0.01 (-0.41,0.40)	CBA
